# Application of Response Surface Methodology for Optimization of the Biosorption Process from Copper-Containing Wastewater

**DOI:** 10.3390/molecules28010444

**Published:** 2023-01-03

**Authors:** Ilona Trawczyńska, Sylwia Kwiatkowska-Marks

**Affiliations:** Department of Chemical and Bioprocess Engineering, Bydgoszcz University of Science and Technology, Seminaryjna 3, 85-326 Bydgoszcz, Poland

**Keywords:** biosorption, copper, wastewater, response surface methodology (RSM)

## Abstract

Copper-containing wastewater is a significant problem in the water industry. In this work, biosorption of copper ions on alginate beads have been considered as a promising solution. The effective diffusion coefficient D_e_ is the parameter describing the diffusion of copper ions in calcium alginate granules. Granules with a wide spectrum of alginate content from several to several dozen percent (0.6–20%) were tested. The granules with an alginate content of 20% were produced by a new method. The conductometric method was used to determine D_e_. The study determined the D_e_ values depending on the process parameters (temperature and pH of copper solutions) and the alginate content in the granules. The RSM method was used to analyze the obtained results. The conducted research proved that all analyzed factors significantly affect the value of the diffusion coefficient (R^2^ = 0.98). The optimum operating conditions for biosorption of copper ions from CuCl_2_ salt, on alginate beads obtained by RSM were as follows: 0.57% of alginate content in the granules, temperature of 60.2 °C, and pH of 2. The maximum value of D_e_ was found to be 2.42·10^−9^ m^2^/s.

## 1. Introduction

Heavy metals can be easily absorbed by marine organisms and crop plants due to their solubility in water and accumulation in the human body [1]. When copper-containing wastewater is discharged into the environment outside the self-purification area, the high toxicity and non-biodegradability of copper ions pose a serious threat to animal and human health. High requirements for the quality of wastewater containing toxic metal ions, produced in various industries, force intensive research into all available methods for their purification [2]. According to WHO recommendations, the maximum allowable concentration of Cu^2+^ in water is 2.0 mg/L [3]. In addition, recovering copper from wastewater also has some economic benefits [4,5].

We selected adsorbtion to treat copper-containing wastewater due to its advantages, such as low initial cost and process simplicity [6,7]. However, the instability of the adsorbents and difficulty in the separation still limit their practical applications. This encourages searching for inexpensive, renewable, and environmentally-friendly biosorbents [8]. Biosorption on materials of natural origin seems to be providing the most prospective results: in addition to it being highly efficient, it enables elimination of the entire content of metal ions, even if they are present at very low concentrations in the liquid waste.

Alginate derived from brown algae is a highly popular material for the biosorption of heavy metals due to its advantages, such as low cost and high affinity via gelation [9,10]. Abundant functional groups have been found in sodium alginate, such as carboxyl and hydroxyl groups, which can crosslink with cations [11,12]. Sodium alginate reacts with divalent cations such as Ca(II), Ba(II) and Sr(II) to form insoluble hydrogels, which are crosslinked to form a reticular structure called the “egg box”, and the crosslinking pathway is the exchange between the sodium ions of α-L-guluronic acid and divalent ions [13,14]. In addition, alginate beads can be easily recovered [15,16]. Consequently, calcium alginate is a promising biomaterial for the biosorption of heavy metals [4,17].

Biosorption of copper ions on alginate beads is influenced by various conditions such as temperature, pH of copper solutions, and the alginate content in the granules [18,19,20]. Optimization of the above-mentioned parameters is imperative in order to obtain high yields of biosorption at lower costs. A classical practice of achieving these is by single variable optimization methods, i.e., varying one factor at a time while keeping others at constant levels. This approach is not only tedious and time-consuming but can also lead to misinterpretation of results, especially because the interaction between different factors is overlooked. Hence, statistical experimental designs by response surface methodology (RSM) may be considered as an efficient way to deal with the limitations of the conventional method [21,22,23]. 

The efficiency of heavy metal removal can be influenced by various factors, such as: temperature, concentration of heavy metals or adsorbents, pH, etc. pH is a very important parameter because pH affects the chemical speciation of metals in solutions, as well as the ionization of chemically active sites on the surface adsorbent [24].

Response surface methodology (RSM) is a combination of statistical and mathematical techniques useful for investigating the interactive effects between several factors at different levels [25]. The experimental workload can be reduced by using statistical design. Among RSM designs, central composite design (CCD) is the most widely used approach for statistical process optimization [26,27,28]. This experimental methodology generates a mathematical model to estimate the connection between the variable and the response variables. So far, no one has studied the usefulness of this method in biosorption of copper ions on alginate beads. Literature data is fragmentary and inconsistent [4,11,18,19,29]. Keeping this in mind, the present work was carried out to optimize different process parameters (temperature and pH of copper solutions) and the alginate content in the granules for the efficient biosorption of the copper on alginate beads using RSM. 

## 2. Results and Discussion

The RSM designs applied in our investigation have been successfully applied in many recent biotechnological applications, however, to the best of our knowledge, no single report was obtained on the optimization of biosorption of copper ions on alginates beads. 

### 2.1. Surface Morphology of Calcium Alginate Beads

**Figure 1:**
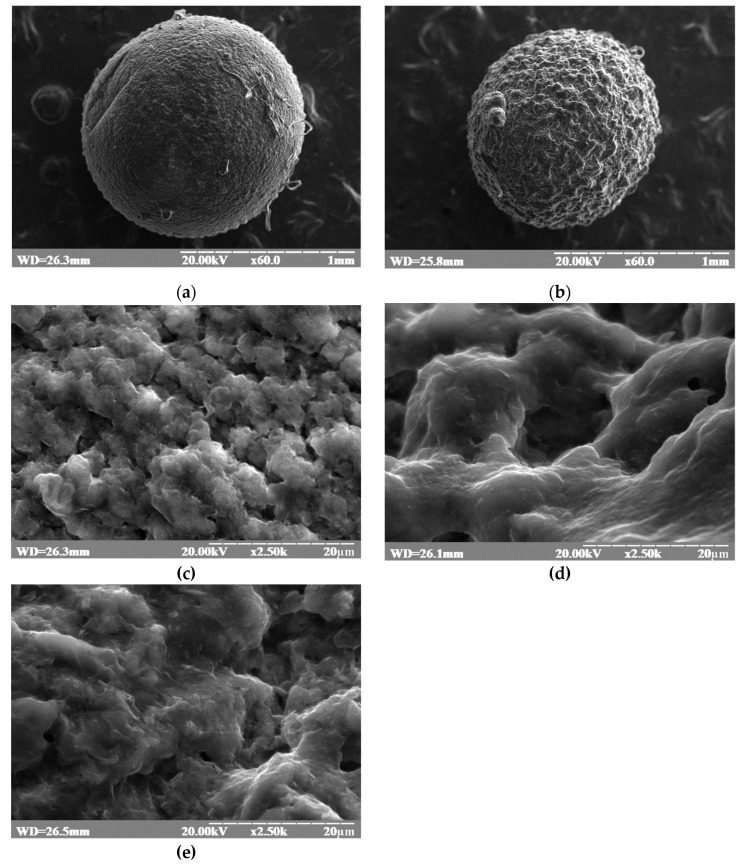
Scanning electron microscopy of calcium alginate beads. (**a**) A total of 4.5% alginate bead without copper, magnified 60 times; (**b**) 20.3% alginate bead without copper magnified 60 times; (**c**) higher magnification (2500 times) of 4.5% alginate bead without copper; (**d**) higher magnification (2500 times) of 20.3% alginate bead without copper (an air pocket in the dark area is visible); and (**e**) 20.3% alginate bead loaded with copper magnified 2500 times.

SEM was used to study the external morphology (size, shape, and surface) of the prepared beads. Randomly selected beads were studied. The images were taken at 60×, and 20,000× magnification. The SEM operating parameters were set at 20 kV to accelerate the voltage. The SEM photographs are depicted in Figure 1, which shows that the beads were almost spherical, with a rough outer surface. SEM analysis showed that the diameter of the beads was 26.3 mm. The photographs presented in Figure 1c,d show differences in the surface morphology of the prepared beads. The surface of 20.3% of the beads was rougher than 4.5% of the beads. Beads with a lower alginate content had a smoother surface. SEM photographs of the blank beads (Figure 1d) compared with copper loaded bead (Figure 1e) also show a difference in surface morphology. Figure 1e indicates that the alginate matrix entrapped copper.

The optimization of the water biosorption ions on alginates beads was carried out to find the optimal values of independent variables (temperature, pH, and alginate content in the granules), which would give maximum effective diffusion coefficient. The results obtained from the different experimental sets are presented in Table 1. On the basis of the CCD, the second order response surface model was obtained from Equation (7).

### 2.2. ANOVA Analysis and the Adequacy of the Mathematical Model

The optimization of the water biosorption ions on alginates beads was carried out to find the optimal values of independent variables (temperature, pH, and alginate content in the granules), which would give a maximum effective diffusion coefficient [25,30,31]. The results obtained from the different experimental sets are presented in Table 1. On the basis of the CCD, the second order response surface model was obtained from Equation (7).

The results of the model in the form of analysis of variance (ANOVA) are given in Table 1. All coefficients in the full quadratic model were analysed with a *T*-test. The larger the magnitude of T-value and smaller the *p*-value, the more significant is the corresponding coefficient. The coefficients β_1_, β_2_, and β_3_ were found to be significant (*p* < 0.05). Therefore, it can be said that the linear terms of all independent variables were significant to the model at 5% level of significance. The final estimative response model equation in terms of effective diffusion coefficient was:

(1) $$Y=1.06+0.31X_{1}-0.18X_{2}-0.18X_{3}-0.05X_{1}^{2}+0.05X_{2}^{2}+0.06X_{3}^{2}-0.02X_{1}X_{2}-0.05X_{1}X_{3}+0.05X_{2}X_{3}$$

**Figure 2:**
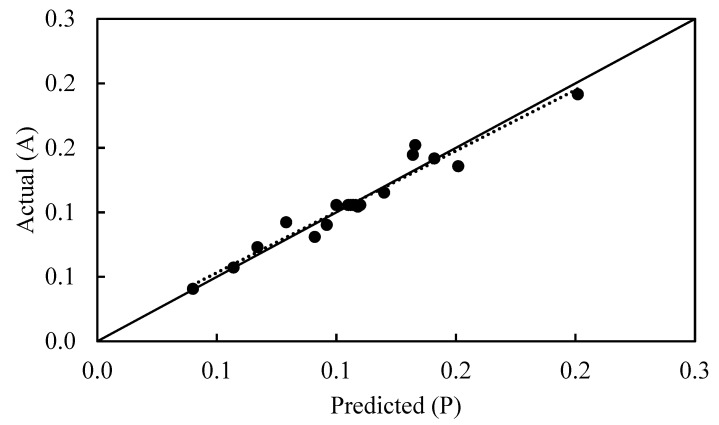
Linear correlation between the actual and predicted effective diffusion coefficient. – A = P, ··· best linear fit, ● data point.

Model validity was confirmed with the coefficient of determination R^2^ values. R^2^ should be 0 < R^2^ < 1 and larger values denote better results. The regression equation obtained indicated an R^2^ value of 0.98. This value ensured a satisfactory adjustment of the quadratic model to the experimental data and indicated that 98% of the variability in the response could be explained by the model. For better evaluation of the model we also used absolute average deviation (AAD) and root mean square error analysis (RMSE) [32]. AAD and RMSE values close to zero are indicative of high accuracy between observed and predicted values. The AAD is calculated by the following equation:

(2) $$\mathrm{AAD}=\left\{\left[\overset{P}{\underset{i=1}{\sum }}\left(\left|Y_{i,\exp}-Y_{i,\mathrm{cal}}\right|/Y\right)\right]/P\right\}\times 100$$

(3) $$\mathrm{RMSE}={\left(\frac{1}{n}\sum_{i=1}^{n}{\left(Y_{i,\exp}-Y_{i,\mathrm{cal}}\right)}^{2}\right)}^{\frac{1}{2}}$$

where Y_i,exp_ and Y_i,cal_ are the experimental and calculated responses, respectively, and P is the number of experimental runs. AAD and RSME values were determined as 0.05 and 0.08, respectively. R^2^ together with AAD and RSME results indicate that the model is sufficient for estimation of the average of effective diffusion coefficient with high accuracy for all experimental points. A satisfactory correlation between experimental and predictive values is also shown by the predicted versus actual plot (Figure 2). The clustered points around the diagonal indicate a good fit of the model.

The predicted optimization result by the model suggested that the maximum effective diffusion coefficient (D_e_ = 2.42·10^−9^ m^2^/s) for biosorption of copper ions on alginates beads could be achieved when temperature, pH, and alginate content in the granules were set at 60.2 °C, 2, and 0.57%, respectively. Test have been conducted [33], in which the maximum D_e_ was also obtained for the lowest alginate content in the granules.

### 2.3. Three-Dimensional Response Surface Plots

**Figure 3:**
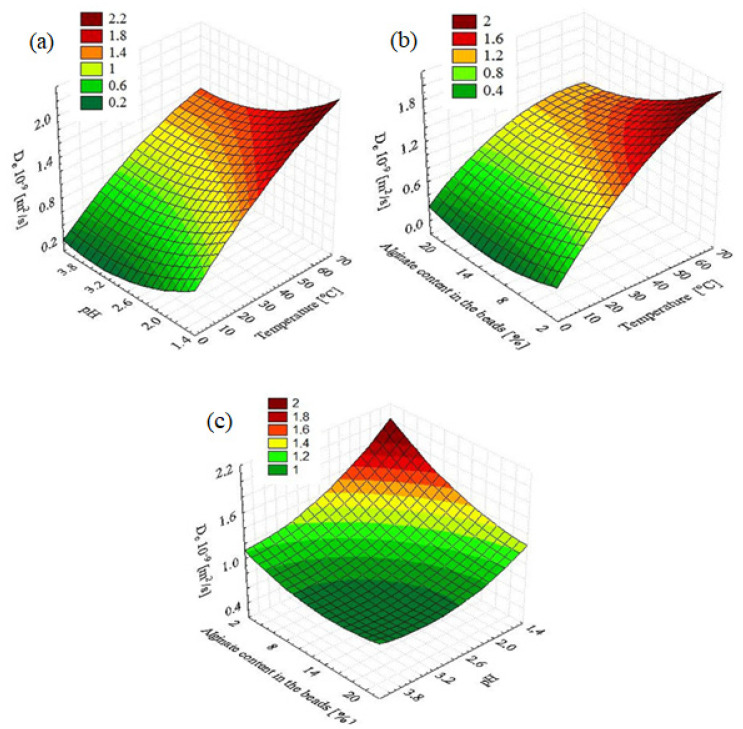
Response surface plots for biosorption process. The effect of (**a**) temperature and pH, (**b**) temperature and alginate content in the beads, and (**c**) alginate content in the beads and pH.

The interaction effects and optimum conditions of the independent variables optimized for enhanced biosorption process are presented by the 3D response surface plots shown in Figure 3. The contour plots were organized based on the quadratic model. Two variables were analyzed at a time while keeping other variables at fixed levels (center point). As is evident from the response surface plot shown in Figure 3a, the increase of temperature and decrease of pH led to corresponding linear increases gradually in the effective diffusion coefficient. The combined effect of temperature and alginate content in the granules is shown in Figure 3b. The effective diffusion coefficient increases for the higher temperature and for the lower concentrations of alginate. The presented research results are consistent with the previously published studies [33]. It was confirmed that the D_e_ coefficient increases with the decrease in the alginate content in the granules.

Figure 3c shows that when both the reaction medium pH and alginate content in the granules decreased, the effective diffusion coefficient increased. Analysis with evaluation of the 3D response surface plots indicated the ranges of the optimum biosorption conditions as follows: temperature, 50–70 °C; alginate content in the granules, 2–6%; and pH of the reaction medium, 1.4–2.2.

The presented research proved that all the analysed factors significantly affect the value of the diffusion coefficient. The new method of obtaining alginate beads made it possible to obtain beads with a high biosorbent content (up to 20% by weight). Due to their different structure, such beads are characterized by lower values of the effective diffusion coefficient (D_e_) compared to beads with an alginate content of a few percentages. Besides the alginate content of the beads, environmental factors such as temperature and pH also influence the (D_e_) value. 

## 3. Materials and Methods

### 3.1. Chemicals

Sodium alginate was purchased from Sigma-Aldrich and calcium(II) chloride from Chempur. Sodium alginic acid was used to fabricate and calcium chloride for crosslinking the alginate beads. CuCl_2_ was purchased from POCH S.A. Avantor Performance Materials Poland SA. To adjust the pH of the solution, 1 M hydrochloric acid (HCl) and 1 M sodium hydroxide (NaOH) were applied, which were acquired from POCH S.A. Interventionary studies involving animals or humans, and other studies that require ethical approval, must list the authority that provided approval and the corresponding ethical approval code.

### 3.2. Preparation of Calcium Alginate Beads

The starting material for the preparation of the beads was a low-viscosity sodium alginate. In spite of the fact that the alginate itself had a low viscosity as compared to that of other alginates, viscosity of its aqueous solutions was so high that the maximum attainable calcium alginate concentration in the beads was only 4.5% wt. Sodium alginate powder was added to the distilled water to obtain a viscous sodium alginate solution. To the obtained spherical biosorbent the aqueous solution of sodium alginate was added drop-wise under gentle stirring, to a 0.05 M CaCl_2_ solution used as a cross-linking medium. The beads were formed immediately. During a 30-min gelation period, Ca(II) ions were bonded to the alginate beads and the sphere became compact. To attain an equilibrium between Ca(II) in solution and the ions adsorbed on the beads, they were placed in a 0.05 M CaCl_2_ solution for 24 h. Alginate beads were stored in a refrigerator in a solution containing 0.01 M of KCl and 0.001 M of CaCl_2_. Before using them, the gel beads were rinsed with distilled water several times to remove free Ca(II).

Producing granules containing a greater concentration of alginate was a problem. The methods so far known have made it possible to get gels of concentrations up to 6.5% wt. The high viscosity of the aqueous solutions of sodium alginate is an obstacle here. A new method of granules preparation containing high concentration of biosorbent has been elaborated and is based on using a sodium alginate suspension. This method is described in detail in Ref. [29]. The beads production started with the preparation of the alginate suspension. Ethyl alcohol of the 96% vol. concentration was used to prepare the suspension. At first, ethyl alcohol was mixed with distilled water at the appropriate proportions. Then, a suitable amount of sodium alginate was added to the previously prepared mixture. The obtained suspension, after careful stirring, was dripped into the crosslinking solution (0.18 M CaCl_2_). Mechanical extrusion was used. The formed beads were maintained for 24 h in the 0.18 M CaCl_2_ solution and then kept in a refrigerator in a 0.01 M KCl solution. Before use, the gel beads were rinsed with distilled water several times. By making use of such a method, three types of the alginate granules with different alginate contents (10.2, 16.0, 19.9% wt.) were produced. The properties all of the obtained granules are listed in Table 2.

Prior to carrying out the measurements of the effective diffusivity by the conductometric method, the calcium(II) ions in the beads were substituted by the copper(II) ions. To do this, an appropriate quantity of the calcium alginate beads (the total volume of the beads of sorbent was 1 mL, to satisfy the condition α ≥ 100) was placed in a 0.1 M CuCl_2_ solution and the mixture was stirred with a magnetic stirrer. After a 2-h saturation period, the beads were transferred to fresh volume of the 0.1 M Cu(II) solution where they were stirred for 24 h. After saturation with Cu(II), the beads shrank in volume by ca. 8%, most likely owing to the exchange of the calcium(II) ions for the copper(II) ones.

### 3.3. Conductometric Method

This method (called conductometric) and the setup for determination of the effective diffusion coefficient De was described in detail by Kwiatkowska-Marks and Miłek [33]. The method was based on carrying out the measurements in a closed system. In a beaker with distilled water, a known quantity of a Cu(II)-saturated alginate was placed and the suspension was vigorously agitated to eliminate the resistance of internal diffusion and to ensure ideal mixing in the system. Under these conditions, Cu(II) ions present in the pores of the sorbent diffuse to the distilled water, with the rate of the process being controlled by effective diffusivity. The increasing Cu(II) concentration in the solution results in the increase in conductance, which is measured with a conductometer. 

Under assumption that the sorbate is uniformly distributed within the whole bead, and that the beads are at equilibrium with the liquid phase, and by selection of the appropriate experimental conditions (α ≥ 100, i.e., the volume of the sorbent is at least 100 times smaller than that of the distilled water), then we can use the equation derived for the open system

(4) $$\frac{C_{t}}{C_{\infty }}=1-\frac{6}{\pi^{2}}\sum_{n=1}^{\infty }\frac{1}{n^{2}}\cdot \exp\left(-\frac{D_{e}n^{2}\pi^{2}t}{R^{2}}\right)$$

where *C_t_* is the sorbate concentration in solution at time *t*, *C*_∞_ is the sorbate’s equilibrium concentration in the solution, *D_e_* is the effective diffusion coefficient, and *R* is sorbent bead radius. 

Because in the new conductometric procedure the determination of the effective diffusivity is based on the measurement of conductance of a solution into which the sorbate diffuses (under assumption of a linear relationship between the conductance and concentration), transformation of the equation for the transient diffusion leads to Equation (5).

(5) $$\frac{P_{t}}{P_{\infty }}=1-\frac{6}{\pi^{2}}\sum_{n=1}^{\infty }\frac{1}{n^{2}}\exp\left(\frac{-D_{e}n^{2}\pi^{2}t}{R^{2}}\right)$$

where *P_t_* is the conductivity of the solution after time *t* and *P*_∞_ is the conductivity of the solution after time ∞.

The apparatus for the determination of the effective diffusivity consists of a 120-cm^3^ beaker, a thermostated water jacket, a magnetic stirrer, a thermometer, and a conductometer with an electrode. 

Conductance of the solution was measured on a microcomputer-assisted CPC-551 (ELMETRON) conductometer. Copper(II)–saturated beads of a volume smaller than 1 cm^3^ were placed in 100 cm^3^ of distilled water, simultaneously starting the magnetic stirrer and a stop-watch. The temperature was held at 25 ± 0.5 °C throughout. Conductance of the solution was measured at set time intervals up to reaching the constant readings on the conductometer (usually within 60 min).

### 3.4. Experimental Design

The research program was designed in such manner that it was possible to obtain the necessary information by performing the least number of analysis possibilities. CCD was used to compare the interactions between the various variables after they were coded. The variables were coded according to the following equation:

(6) $$X=\frac{x-\left(x_{\max}+x_{\min}\right)/2}{\left(x_{\max}-x_{\min}\right)/2}$$

where x is the natural variable, X is the coded variable, and x_max_ and x_min_ are the maximum and minimum values of the natural variable [30,31].

Each factor was examined at five different levels coded as shown in Table 3. For the three independent variables, i.e., reaction medium temperature (X_1_), reaction medium pH (X_2_), and alginate content in the beads (X_3_), CCD is composed of 20 experiments. The design includes eight basis points that are a single run for each of the −1 and 1 level combinations, six replications of the center points (all factors at level 0), and the six star points, that is, points having one factor for the axial distance to the center of ±α, whereas the other two factors are at level 0. The axial distance α was chosen to be 1.682 to make this design orthogonal. The experimental design used for the study is shown in Table 4. All the experiments were done in triplicate and the average of effective diffusion coefficients D_e_·10^−9^ [m^2^/s] obtained was taken as the dependent variable or response (Y).

Fitting the experimental data can be described by using a second-order polynomial response surface model:

(7) $$=\beta_{0}+\overset{3}{\underset{i=1}{\sum }}\beta_{i}X_{i}+\overset{3}{\underset{i=1}{\sum }}\underset{j>i}{\sum }\beta_{\mathrm{ij}}X_{i}X_{i}+\overset{3}{\underset{i=1}{\sum }}\beta_{\mathrm{ii}}X_{i}^{2}+\varepsilon$$

where Y is the predicted response (the average of effective diffusion coefficient), terms of β_0_, β_i_, β_ii_, β_ij_, and ε illustrates the offset term, the linear effect, the squared effect, the interaction effect, and the residual term, respectively. X_i_ and X_j_ represent the coded independent variables [30,31]. The predicted polynomial model was analyzed using the response surface regression procedure. The coefficients of Equation (7) were determined using STATISTICA software.

### 3.5. Determination of the Point of Zero Charge (pH_pzc_) 

The point of zero charge (pH_pzc_) is defined as the pH of the solution at which the charge of the positive surfaces is equal to the charge of the negative surfaces, i.e., the surface charge of the adsorbent is zero [34]. The surface charge is negative at pH > pH_pzc_ and positive at pH < pH_pzc_ [35].

The zero-charge point (pH_pzc_) of the solid adsorbents was determined by 50 mL of 0.01 M NaCl solutions (being the background electrolyte), which were placed in several closed Erlenmeyer flasks. The initial pH (pH_i_) in each flask was adjusted to a range of 2 to 12 by adding HCl (0.1 M) or NaOH (0.1 M) solutions. The pH was measured with a pH meter. An aliquot of the sample (0.1 g) was then added to each flask. The flasks were shaken at 300 rpm for 24 h at room temperature. The final pH (pH_f_) was then measured. pH_pzc_ is the point where pH_f_ − pH_i_ = zero. The pH_pzc_ obtained for the alginate beads was 6.3 and is comparable to the literature (6.2) [36].

## 4. Conclusions

The RSM method can be used to optimize the sorption of copper ions on calcium alginate granules. Based on the research, a conclusion can be drawn, stating that the increase in the temperature of the copper sorption process on the alginate biosorbent increases the value of the effective coefficient of copper ion diffusion in the granules. Since sorption of Cu (II) on calcium alginate granules occurs better at acidic pH, it was decided that the tested pH range would from 1.5 to 4. In the tested range, an increase in pH caused a decrease in the D_e_ value. Regardless of the process temperature, the highest D_e_ value was obtained for the lowest pH. Therefore, the sorption of Cu(II) ions is recommended to be carried out at the highest possible temperature and the lowest pH. Under these conditions it is possible to obtain the highest D_e_, and it is therefore the most efficient part of the process.

## Figures and Tables

**Table 1:** ANOVA for the second order response surface model.

Term	Coefficient	SE Coefficient	T, DF = 10	*p*-Value
$\beta_{0}$	1.06	0.04	21.56	0.00
$\beta_{1}$	0.31	0.03	9.50	0.00
$\beta_{2}$	−0.18	0.03	−5.66	0.00
$\beta_{3}$	−0.18	0.04	−4.46	0.01
$\beta_{1}^{2}$	−0.05	0.03	−1.42	0.19
$\beta_{2}^{2}$	0.05	0.03	1.69	0.12
$\beta_{3}^{2}$	0.06	0.04	1.43	0.18
$\beta_{12}$	−0.02	0.04	−0.44	0.66
$\beta_{13}$	−0.05	0.04	−1.26	0.23
$\beta_{23}$	0.05	0.04	1.09	0.30

R = 0.95; R^2^ = 0.98; R^2^_(adjusted)_ = 0.92; SE coefficient: standard error of coefficient; T: test coefficient; DF: degree of freedom; *p*: probability value.

**Table 2:** Characteristics of the obtained alginate beads.

Mass of Alginate [g]	Mass of Ethyl Alcohol [g]	Mass of Water [g]	Alginate Content in Beads [% wt.]	Diameter of the Beads [mm]
5	0	95	4.5	2.9
4	12	36	10.2	2.4
7	12	36	16.0	2.2
10	12.5	36	19.9	2.2

**Table 3:** Decoding values of independent variables used in the experimental design.

Variables	Decoding Value				
	−α	−1	0	1	+α
**Temperature [°C]**	9.8	20	35	50	60.2
**pH**	1.5	2	2.75	3.49	4
**Alginate content in the beads [%]**	0.57	4.5	10.25	16	19.92

**Table 4:** Experimental design and results of the CCD.

Run Order	Independent Variables			Y
	X_1_	X_2_	X_3_	
1	−1	−1	−1	1.20
2	−1	1	−1	0.67
3	−1	−1	1	0.91
4	−1	1	1	0.57
5	1	−1	−1	2.01
6	1	1	−1	1.41
7	1	−1	1	1.51
8	1	1	1	1.09
9	1.682	0	0	1.32
10	−1.682	0	0	0.4
11	0	1.682	0	0.96
12	0	−1.682	0	1.33
13	0	0	1.682	0.79
14	0	0	−1.682	0.52
15	0	0	0	1.07
16	0	0	0	1.06
17	0	0	0	1.08
18	0	0	0	1.05
19	0	0	0	1.00
20	0	0	0	1.10

## Data Availability

Not applicable.
